# Skull base ligamentous mineralisation: evaluation using computed tomography and a review of the clinical relevance

**DOI:** 10.1186/s13244-019-0740-8

**Published:** 2019-05-21

**Authors:** Philip Touska, Sultana Hasso, Alp Oztek, Fungayi Chinaka, Steve E. J. Connor

**Affiliations:** 1 Department of Radiology, Guy’s and St. Thomas’ NHS Foundation Trust, 2nd Floor Tower Wing, Guy’s Hospital, Great Maze Pond, London, SE1 9RT UK; 2 Department of Radiology, University of Washington Medical Center, 1959 NE Pacific St, Seattle, WA 98195 USA; 3 Department of Neuroradiology, King’s College Hospital NHS Trust, Denmark Hill, Brixton, London, SE5 9RS UK; 4 School of Biomedical Engineering and Imaging Sciences Clinical Academic Group, King’s College London, King’s Health Partners, Guy’s Hospital, London, UK

**Keywords:** Skull base, Ligaments, Ossification, Pterygoid muscles, Mandibular nerve, Carotid artery, internal

## Abstract

**Objectives:**

To determine the frequency, morphologic and demographic characteristics, and clinical relevance of the mineralisation of six skull base ligaments (interclinoid, caroticoclinoid, petrosphenoid, posterior petroclinoid, pterygospinous, and pterygoalar).

**Methods:**

This is a retrospective review of 240 CT scans of the paranasal sinuses (ages 6–80 years). A limited systematic review was performed primarily using Embase and Medline databases.

**Results:**

Ligamentous mineralisation was well delineated on CT and occurred at ≥ 1 location in 58.3% of patients. There was a nonsignificant trend towards a greater incidence with advancing age. The interclinoid and posterior petroclinoid ligaments were most commonly mineralised (22.1% and 18.3%, respectively); the petrosphenoid and pterygoalar ligaments were least frequently mineralised (10.8% and 6.3%, respectively). The mean age of patients with posterior petroclinoid mineralisation was significantly greater than those with interclinoid and petrosphenoid mineralisation and was not seen in patients aged 6–20 years. The literature review highlighted the clinically relevant potential for mineralised ligaments to cause barriers to surgical access (e.g. to the foramen ovale), increase the risk of neurovascular injury during surgery at the skull base (e.g. during anterior clinoidectomy), and predispose to neural impingement.

**Conclusions:**

Skull base ligamentous mineralisation is commonly encountered on CT imaging. Given the potentially significant clinical implications, an understanding of the morphological appearances is of importance to those planning interventions at the skull base. To the authors’ knowledge, this study is the first to comprehensively evaluate such a wide range of skull base ligaments using CT. For some ligaments, the incidence on CT has not been previously described.

## Key points

- Skull base ligamentous mineralisation is common and seen in most age groups, aside from the posterior petroclinoid ligament, which is has a stronger association with age, reflecting its dural origin.
- Mineralisation of the interclinoid and caroticoclinoid ligaments can increase the risks of several surgical procedures at the skull base (including during the treatment of aneurysms). Knowledge of such structures is important in operative planning.
- Ossified ligaments have been associated with neural impingement syndromes of the abducens nerve (petrosphenoid ligament), oculomotor nerve (petroclinoid ligament), and mandibular nerve branches (pterygospinous and pterygoalar ligaments).

## Introduction

Several ligaments exist at the skull base, but knowledge of their anatomy is limited amongst clinicians owing to the paucity of coverage in mainstream anatomical texts. However, improvements in minimally invasive neurosurgical techniques have made accurate identification of these structures invaluable for surgical planning, particularly when they become mineralised [1–3]. Mineralised ligaments can present barriers to surgical access, alter the appearances of familiar anatomical landmarks, or prevent structural mobilisation during surgery, thereby increasing the risk of neurovascular injury [4–6]. Additionally, mineralised skull base ligaments have been implicated in neural impingement syndromes as a result of mechanical compression of nerves against ossified bars or within the foramina that mineralised ligaments may form [7–11]. Hence, skull base ligamentous ossification is relevant to radiologists, neurologists, and neurosurgeons managing patients with skull base pathology.

The available literature is predominantly derived from studies of dry skulls, with only a minority using imaging to evaluate these structures (see the tabulated summary of the subsequent systematic review). To the authors’ knowledge, this represents the first comprehensive study to use computed tomography (CT) to systematically evaluate the frequency of incidental skull base ligamentous mineralisation in a modern ethnically diverse population.

## Materials and methods

**Fig. 1:**
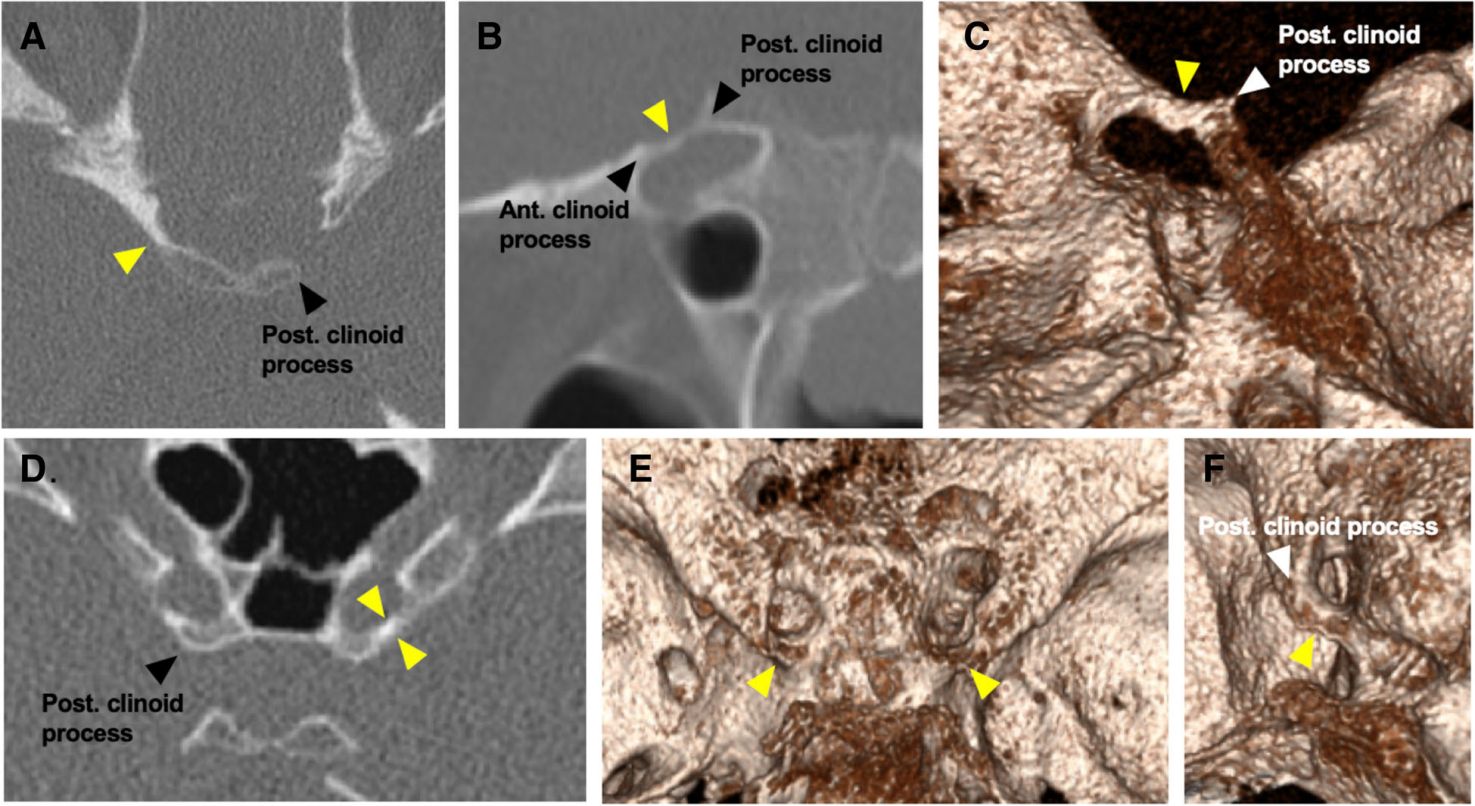
Mineralised caroticoclinoid and interclinoid ligaments. Axial (**a**), sagittal oblique (**b**), and 3D (**c**) volume reconstruction demonstrating a complete interclinoid ligament on the right (yellow arrowhead). Axial CT (**d**) and 3D (**e**) volume reconstructions demonstrating bilateral complete caroticoclinoid ligaments (yellow arrowheads). **f** 3D reconstruction demonstrating the right-sided complete caroticoclinoid ligament (yellow arrowhead)

**Fig. 2:**
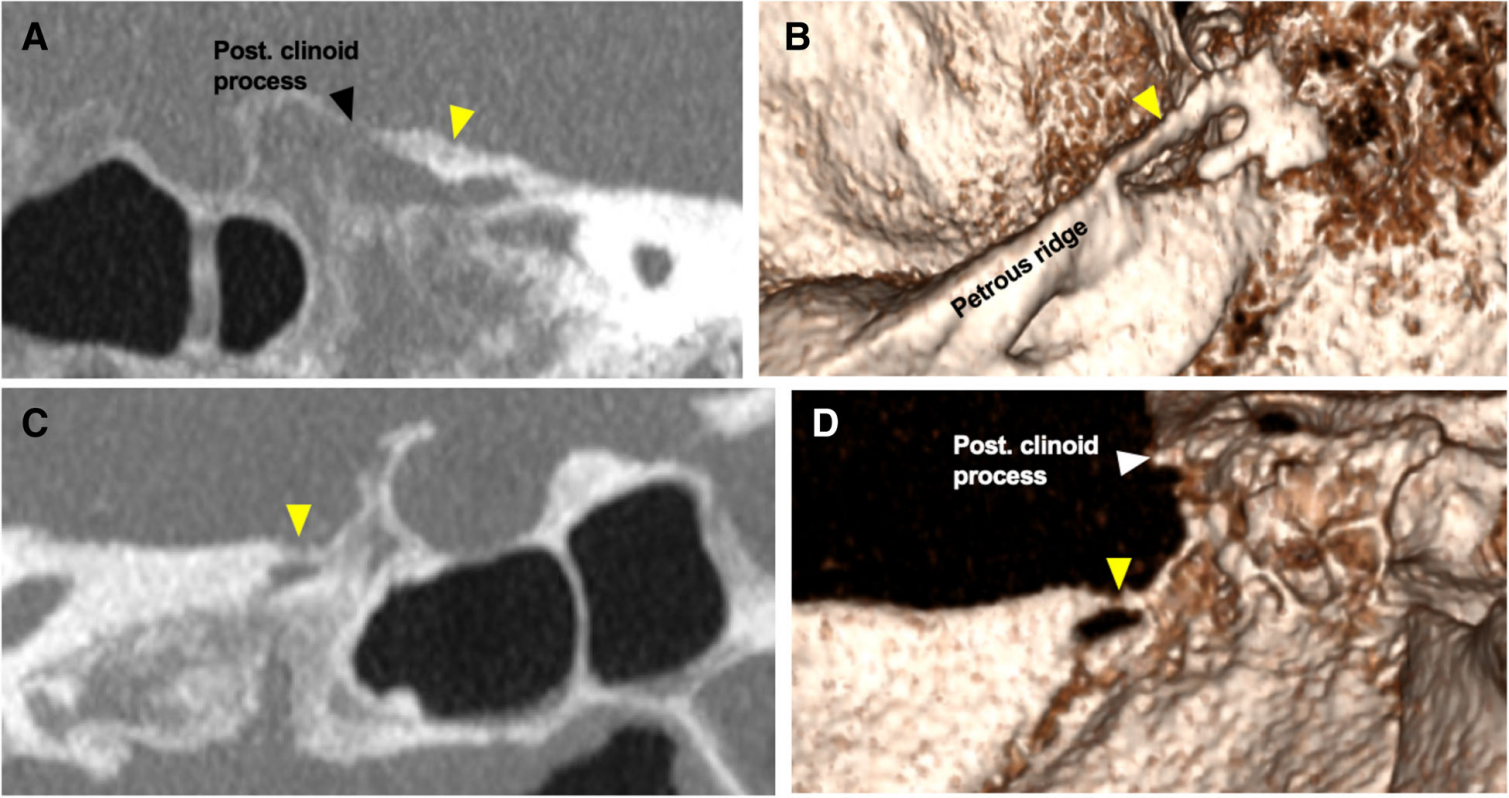
Mineralised posterior petroclinoid and petrosphenoid ligaments. **a** Oblique-sagittal maximum-intensity projection (MIP). **b** 3D volume reconstruction of a right-sided, completely mineralised posterior petroclinoid ligament (or dural fold) (yellow arrowhead). **c** Oblique-sagittal maximum-intensity projection (MIP). **d** 3D volume reconstruction of a right-sided, complete petrosphenoid bar (yellow arrowhead)

**Fig. 3:**
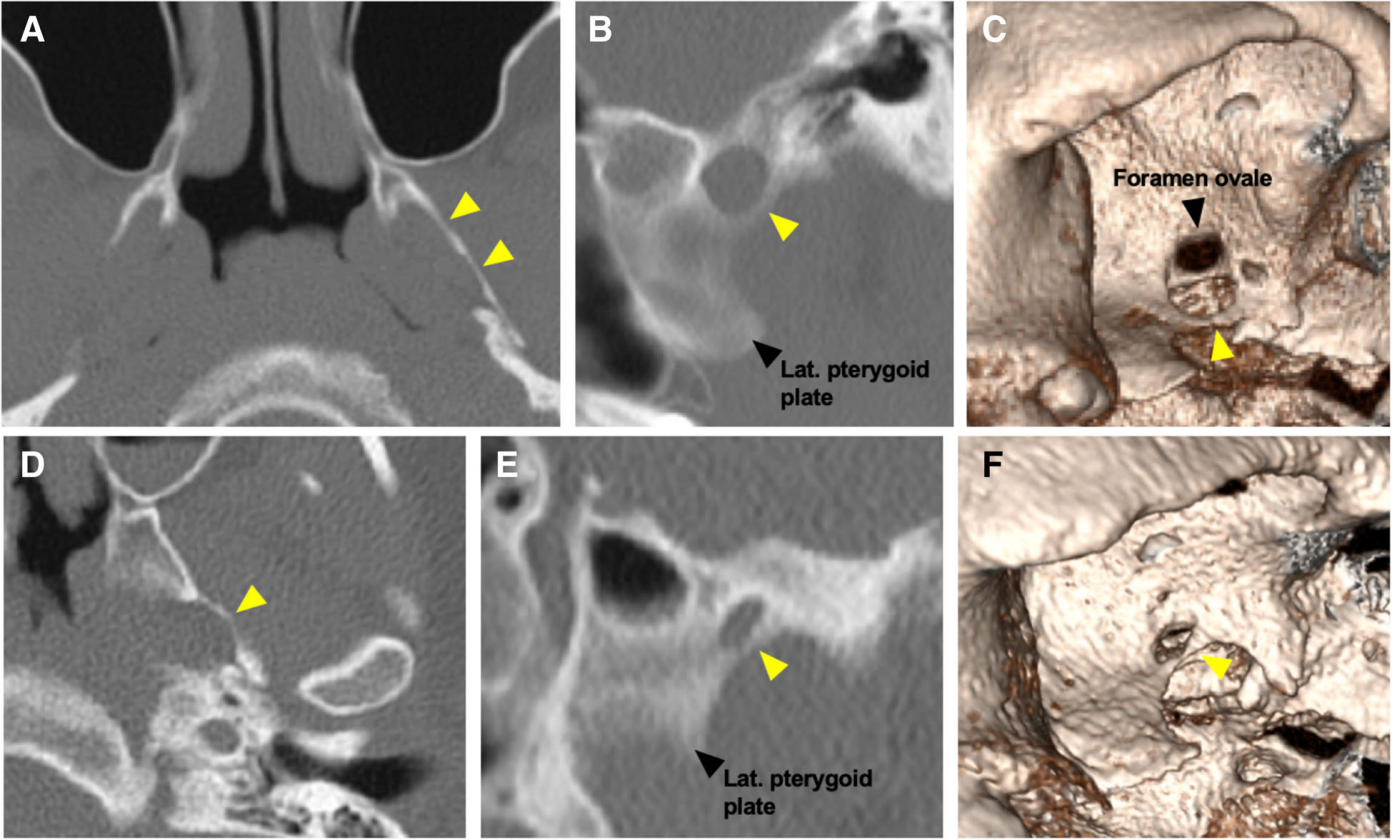
Mineralised pterygospinous and pterygoalar ligaments. Axial (**a**), oblique sagittal MIP (**b**), and 3D (**c**) volume CT reconstructions demonstrating a completely mineralised left pterygospinous ligament (yellow arrowheads). The foramen ovale is demonstrated on the 3D reconstruction. Axial (**d**), oblique sagittal MIP (**e**), and 3D (**f**) volume CT reconstructions demonstrating a completely mineralised left pterygoalar ligament (yellow arrowheads). The foramen ovale is obscured

Institutional approval was obtained, and the requirement for informed consent waived. A retrospective review of high-resolution, non-contrast CT studies of the paranasal sinuses (scanned between April 2014 and January 2017) was carried out. Consecutive cases were selected until equal numbers were achieved for each of 15 age groups (range 6–80 years). Scanning took place on a variety of systems, including SOMATOM Definition Edge (Siemens Healthcare, Erlangen, Germany), iCT, and Brilliance 40 (Philips Medical Systems, Eindhoven, Netherlands) scanners using a kVp of 120 kV, mAs of 25-50, minimum collimation of 0.6–0.625 mm, and a pitch of 0.624–0.8. Each imaging study was evaluated by there independent observers PT, SH, and FC, and the presence of mineralisation (calcification or ossification) for the six skull base ligaments was recorded. Initial detection was carried out by analysing thin axial reconstructions, and detailed evaluation of morphology was carried out using multiplanar reconstructions. The ligaments examined, their anatomical courses, and planes used to evaluate them on CT are detailed in Table 1. Examples of the appearances of the ligaments on CT are demonstrated in Figs. 1, 2, and 3.

**Table 1:** Ligament characteristics

Ligament	Course	Plane used for evaluation
Interclinoid ligament	Extends between the anterior and posterior clinoid processes (or occasionally middle and posterior clinoid processes). When completely mineralised, it can form a common interclinoid foramen [12, 13].	Double oblique sagittal
Caroticoclinoid (anterior interclinoid) ligament	Extends between the anterior and middle clinoid processes. When completely mineralised, it forms the clinocarotid canal traversed by the ICA [12, 14]	Double oblique axial
Petrosphenoid (Grüber’s) ligament	Extends from the petrous tubercle (medial to the trigeminal impression) at the petrous apex to the lower aspect of the posterior clinoid process [15–17].	Double oblique sagittal
Posterior petroclinoid ligament	Extends from the petrous ridge to the posterior clinoid process [18, 19].	Double oblique sagittal
Pterygospinous (Civinini) ligament	Extends from the spine of the sphenoid to the posterior aspect of the lateral pterygoid plate. When completely mineralised, it forms the foramen of Civinini [20–22].	Double oblique sagittal
Pterygoalar (Hyrtl-Calori or ‘innominate’) ligament	Extends from the root of the lateral plate of pterygoid process to the infratemporal surface of the greater sphenoid wing, lateral to the foramen spinosum. Historically, complete mineralisation of the pterygoalar ligament was termed the porus crotaphiticobuccinatorius of Hyrtl (derived from the historic term for the mandibular nerve with deep temporal—or crotaphitic—and buccinator branches) [20–22].	Double oblique sagittal

In each case, mineralisation was considered ‘partial’ if it extended from 50 to < 100% of the ligament’s length and ‘complete’ if it extended to involve the entire length of the ligament. The so-called contact type of mineralisation, where a subtle suture line may be seen at the midpoint of an osseous bar, was considered complete for the purposes of this study [12]. If complete mineralisation resulted in the formation of a foramen, the thickness of the bony bar (at its midpoint) and the corresponding foraminal area were measured using double oblique sagittal reformats on a PACS workstation using syngo.via software (Siemens Healthcare, Erlangen, Germany). Ligaments with < 50% mineralisation, including small bony spurs, were excluded. The use of 50% was chosen as it was felt to be both clinically relevant and simpler to facilitate reproducibility; it has also been employed in prior studies of ligamentous mineralisation [18, 23]. In the case of interobserver discordance, an agreement was achieved through consensus. Where available, demographic information was recorded.

Statistical testing of multiple correlated samples was carried out using a one-way ANOVA with post hoc analysis using the modified Tukey method and two-tailed *t* testing, and the chi-squared test was employed to analyse the distribution of categorical variables using Vassarstats [24] and Microsoft Excel® (Redmond, WA); a *p* value of < 0.05 was deemed to be significant.

A systematic review of the English language literature was carried out as per PRISMA [25] guidelines using Embase and Medline databases primarily with additional studies identified through study references and a limited search using Google Scholar. The following search terms were utilised ‘interclinoid’, ‘caroticoclinoid’, ‘sellar bridge’, ‘petrosphenoid’, ‘petroclinoid’, ‘pterygospinous’ ‘Civinini + ligament’, ‘pterygoalar’, ‘Hyrtl + ligament’, and ‘crotaphitico-buccinatorius’. Studies were excluded if they were deemed irrelevant (e.g. pertaining to other parts of the body). Selected case reports were included if a potentially clinically consequential observation was documented.

## Results

### Demographics

A total of 240 CT studies were reviewed comprising 121 female (50.4%) and 119 male (49.6%) patients. The patients were divided into 15 groups according to age, with each group spanning 5 years (e.g. 6–10 years). The average age was 42.7 years (range 6–80 years). The majority of patients were white British/European (62.5%; *n* = 150) followed by black British/African and Caribbean (18.3%; *n* = 44), Southeast Asian (Indian subcontinent) (11.3%; *n* = 27), and a group comprising Middle Eastern, East Asian (Chinese), mixed ethnicity, and other/unknown ethnicity (7.9%; *n* = 19).

Partially or completely mineralised skull base ligaments in at least one location were found in 58.3% of patients (*n* = 140).

**Fig. 4:**
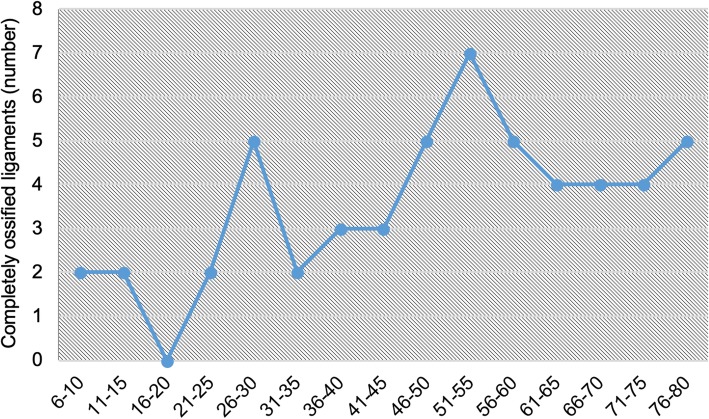
Frequency of complete ligament ossification amongst different age groups

**Fig. 5:**
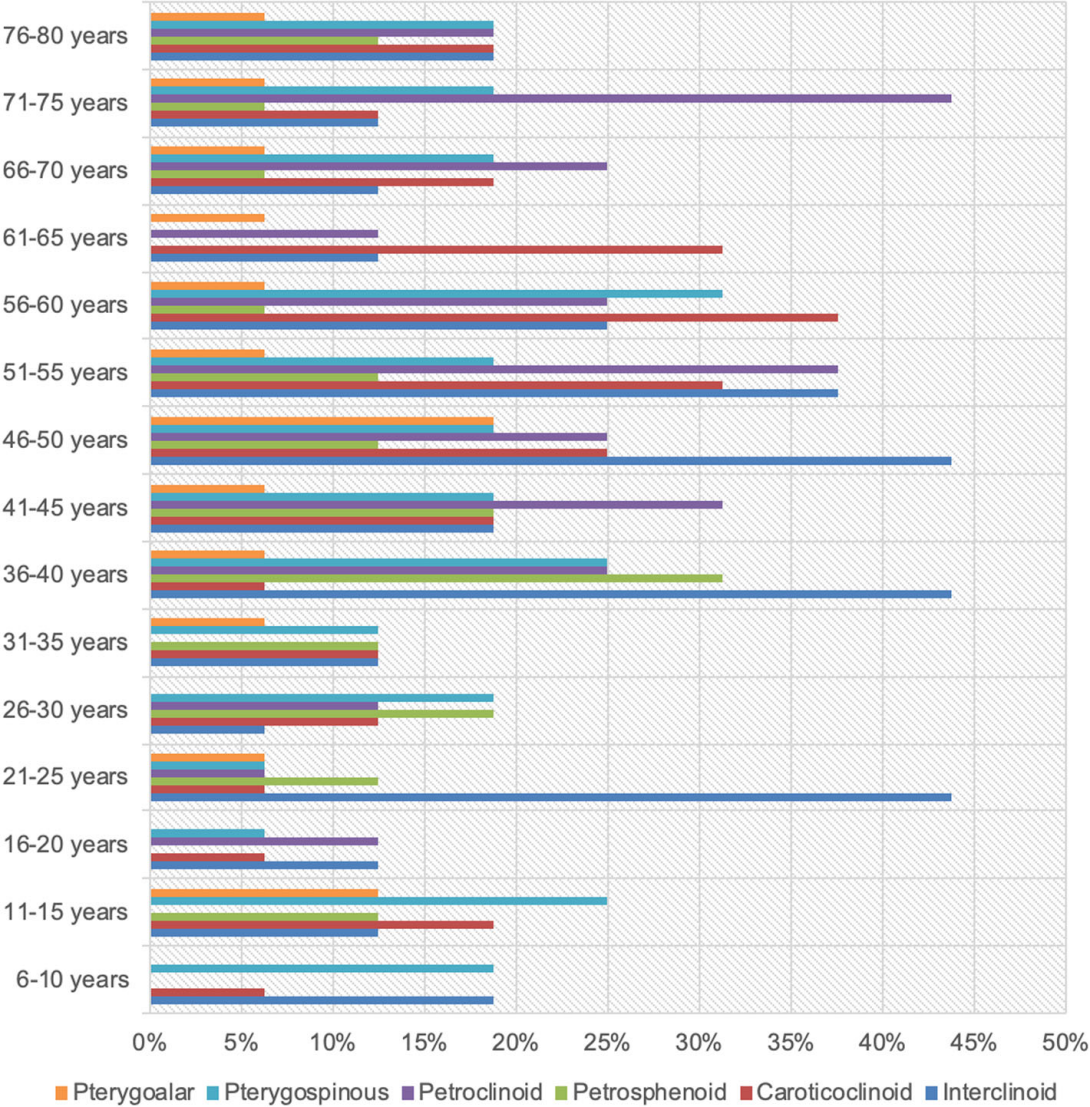
Ligament ossification by type and age group

Mineralisation was observed in all age groups, but least frequently amongst the 16–20 years age group (31%) and most frequently in the 56–60 years age group (81%) Fig. 4. Dividing the population into 5 larger groups of 48 patients, each revealed lower mean proportions of mineralised ligaments amongst the 6–20 and 21–35 years groups compared with older patients. Although the difference was nonsignificant (*p* = 0.0795, using a one-way ANOVA test), there was a trend towards increasing mineralisation with age. Additionally, the rate of complete mineralisation (patients with ≥1 completely ossified bar on either side) showed increasing frequency with age Fig. 5. The mean proportion of patients with at least 1 completely ossified ligament (*n* = 53) were as follows: 6–20 years = 4%, 21–35 years = 9%, 36–50 years = 11%, 51–65 years = 16%, and 66–80 years = 13%. The difference between the means was significant (one-way ANOVA: F-ratio = 4.06; *p* = 0.0329); however, on breakdown of the differences between the means using the Tukey method, only the difference between the 6–20 year and 51–65 year groups was found to be statistically significant (*p* = < 0.05).

The proportion of patients with mineralised ligaments was highest amongst those of white British/European heritage, followed by black British, African, and Caribbean heritage (57%) and those of British Asian/Southeast Asian heritage (52%). The lowest proportion was seen amongst those of other heritages. However, the difference between the proportions of ossified ligaments amongst white and black and white and Southeast Asian patients was nonsignificant (*p* = 0.635 and *p* = 0.382, respectively, using a chi-squared test).

Overall, there was a very slight male preponderance for ligamentous mineralisation with 74 males and 66 females (M:F = 1.12:1).

### Ligament type

The incidence of ligamentous ossification (both partial and complete) varied according to the ligament type, with the interclinoid ligament being most commonly identified and the pterygoalar ligament least commonly identified (the proportions for all ligaments are detailed in Table 2).

**Table 2:** Ossification of ligament types (in descending order of frequency)

	Total (*n*)	Proportion (%)
Interclinoid	53	22.1
Petroclinoid	44	18.3
Caroticoclinoid	42	17.5
Pterygospinous	41	17.1
Petrosphenoid	26	10.8
Pterygoalar	15	6.3

The majority (four of six) of mineralised ligaments were more commonly unilateral, but the caroticoclinoid and petroclinoid ligaments were more commonly bilateral. The proportions of bilaterally and unilaterally mineralised ligaments are detailed in Table 3.

**Table 3:** Characteristics of mineralised ligaments

	Bilateral					Unilateral			
	Total		Complete (%)	Mixed* (%)	Partial (%)	Total		Complete (%)	Partial (%)
	%	*n*				%	*n*		
Interclinoid	45.3	24	29.2	12.5	58.3	54.7	29	20.7	79.3
Caroticoclinoid	59.5	25	60.0	24.0	16.0	40.5	17	52.9	47.1
Petrosphenoid	26.9	7	14.3	0.0	85.7	73.1	19	26.3	73.7
Petroclinoid	56.8	25	4.0	4.0	92.0	43.2	19	10.5	89.5
Pterygospinous	34.1	14	7.1	42.9	50.0	65.9	27	14.8	85.2
Pterygoalar	20.0	3	33.3	66.7	0.0	80.0	12	16.7	83.3

*Mixed cases were those in which where complete mineralisation occurred only on one side

Mineralised interclinoid and caroticoclinoid ligaments could be seen in all age groups. However, the remaining ligament types were not present in all age groups; for example, mineralised petrosphenoids were not encountered in the 6–10 and 16–20 year groups. The frequencies of each ligament type amongst the various age groups are depicted in Fig. 3.

Overall, there was no statistically significant difference between the mean ages of patients with mineralised ligaments (0.0777, using a one-way ANOVA test); however, breakdown analysis of the differences between the groups revealed a significantly higher mean age for patients with posterior petroclinoid ligamentous mineralisation compared to those with interclinoid and petrosphenoid mineralisation (*p* = 0.004 and *p* = 0.009, respectively).

The thickness of the mineralised ligaments varied slightly, with the thinnest being the pterygospinous (Table 4). The smallest foramen was formed by the mineralised petrosphenoid ligament, and the largest foramen was formed by the mineralised interclinoid ligament (Table 4).

**Table 4:** Ligament thickness and foramen size

	Ligament thickness		Mean foramen size		
	Mean (mm)	SD (mm)	Mean (mm^2^)	SD (mm^2^)	Range (mm^2^)
Interclinoid	1.8	0.5	75.2	22	43–142
Caroticoclinoid	1.8	0.8	25.3	4.4	24–40
Petrosphenoid	1.1	0.5	7.2	5.5	2–18
Petroclinoid	1.3	0.5	39	14.5	24–60
Pterygospinous	0.95	0.3	39.9	32.9	2–112
Pterygoalar	1.2	0.4	13.3	7.2	4–25

### Multiple ligaments

Ossification of multiple (> 1) ligament types was observed in 26.7% (*n* = 64) patients. The majority (76.6%; *n* = 49) of these patients had a combination of two ossified ligaments, with the interclinoid and caroticoclinoid ligaments in combination (*n* = 20) and the petroclinoid and pterygospinous ligaments in combination (*n* = 11) being the most common. Ossification of > 2 ligament types was seen in 23.4% (*n* = 15) of patients and ossification of > 3 ligament types in 3.1% (*n* = 2) of cases.

### Limited systematic review

Screening yielded 492 abstracts in the initial search; however, following the removal of duplicates and studies that did not meet the inclusion criteria, 61 records remained for inclusion (Table 5).

**Table 5:** Systematic review

Author	Number included	Population	Age range	Mineralisation			Significance
				Partial	Complete	Both	
Caroticoclinoid ligament							
Current study	240 CT studies	UK	6–80 years	5%	10%	17.5% (includes mixed 2.5%)	–
Archana et al. [26]	250 dry skulls	India	–	6.80%	5.20%**	12%	Neurosurgical implications.
Boyan et al. [27]	34 dry skulls	Turkey	Adults			35.3%	Neurosurgical implications.
Brahmbhatt et al. [28]	50 dry skulls	India	Adults	Not assessed (complete only)	2/50 skulls (4%)	–	Need for awareness amongst radiologists and neurosurgeons.
Dagtekin et al. [29]	15 cadaveric heads + 25 dry skulls	Turkey	–	10%	15%	–	Neurosurgical implications.
Efthymiou et al. [30]	76 dry skulls	Greece	Adults	69.3% (of ossified ligaments)—equivalent of 46% of total	30.7% (of ossified ligaments)—equivalent of 20.4% total**	74%	Neurosurgical implications.
Erturk, Kayalioglu, and Govsa [31],	119 dry skulls + 52 cadaveric heads	Turkey	–	14.91	8.77%**	35.67%	Neurosurgical implications and relationship with cavernous sinus.
Fernandez-Miranda et al. [6]	100 CT angiograms + 50 anatomic specimens	USA	–	–	20%	–	Importance with respect to endonasal neurosurgery.
Gibelli et al. [32]	300 CT head scans	Italy	18–99 years	–	8.70%	–	Association between interclinoid and caroticoclinoid bridging. No association with age or sex.
N. Gupta, Ray, and Ghosh [33]	35 dry skulls	Nepal	–	11.40%	8.60%**	20%	Neurosurgical implications.
Keyes 1935 [12]	2187 dry skulls	USA	1 day–105 years	–	–	34.84%	Details of the anatomical features of mineralised ligament. Complete ossification present in cases as young as 21 days.
Kapur and Mehić [34]	200 dry skulls	Bosnia and Herzegovina	19–91 years	9.75%	7%**	16.75%	Neurosurgical implications.
Lee et al. [35]	73 dry skulls	Korea	n/s	11.60%	4.10%	15.7%	Neurosurgical implications.
Miller, Chamoun, and Beahm [3]	150 maxillofacial CTs	USA	–	–	41.80%	–	Neurosurgical implications for expanded endoscopic approaches.
Natsis et al. [14]	123 dry skulls	Greece	20–91 years	36.60%	23.60%	60.16%	Association between complete mineralisation and age and bilaterality.
Ota et al. [2]	72 CT angiograms for paraclinoid aneurysms	Japan	–	–	–	16.60%	Use of preoperative CT prior to extradural anterior clinoidectomy.
Peker et al. [8]	80 dry skulls	Turkey	–	–	–	34.2%	Neurosurgical implications.
Sharma et al. 2018 [5]	2726 dry skulls	USA	18–105 years	42%*	31%**	–	Neurosurgical implications including risks of injury to the internal carotid artery.
Skrzat, Mroz, and Marchewka [19]	80 dry skulls	Poland	Adults	–	16.3%	–	Neurosurgical implications and effects upon the internal carotid artery.
Suprasanna and Kumar [36]	54 CT angiograms	India	18–70 years	–	–	22.20%	Importance of imaging in pre-operative planning in treating paraclinoid aneurysms.
Aggarwal, Gupta, and Kumar [37]	67 dry skulls	India	–	13.4%	3.0%	16.4%	Neurosurgical implications.
Interclinoid ligament							
Current study	240 CT studies	UK	6–80 years	15.4%	5.4%	22.1% (includes mixed 1.3%)	–
Archana et al. [26]	250 dry skulls	India	–	2.40%	1.60%**	4%	Neurosurgical implications.
Boyan et al. [27]	34 dry skulls	Turkey	Adults	5.9%	5.9%	11.8%	Neurosurgical implications.
Brahmbhatt et al. [28]	50 dry skulls	India	Adults	–	2% (1/50 skulls)	–	Need for awareness amongst radiologists and neurosurgeons.
Cederberg et al. [23]	255 lateral cephalometric radiographs	USA	8–76 years	38.4%	8.2%	–	Weak association between advancing age and degree of mineralisation.
Dagtekin et al. [29]	15 cadaveric heads + 25 dry skulls	Turkey	–	–	5%	–	Neurosurgical implications.
Erturk, Kayalioglu, and Govsa [31]	119 dry skulls + 52 cadaveric heads	Turkey	–	–	8.18%	–	Neurosurgical implications and relationship with cavernous sinus.
Gibelli et al. [32]	300 CT head scans	Italy	18–99 years	–	16.00%	–	Association between interclinoid and caroticoclinoid bridging. Potential association with interclinoid mineralisation and age.
Gupta et al. [38]	1	India	–	–	–	–	Case report—misidentification of a mineralised interclinoid ligament as para-posterior communicating artery aneurysm.
Keyes 1935 [12]	2187 dry skulls	USA	1 day–105 years	–	–	8.68%	Details of the anatomical features of mineralised ligament. Complete mineralisation in cases as young as 6.
Kucia et al. [39]	322 lateral cephalograms	Poland	8–16 years	–	–	11.80%	Possible association with malocclusion.
Leonardi et al. [40]	34 dry skulls	Italy	8–16 years	33.7% (controls); 58.8% (cases)	9.9% (controls); 17.6% (cases)	–	Higher incidence of sellar bridge formation in patients with dental anomalies.
Marşan et al. [41]	118 lateral cephalograms	Turkey	Adult females (mean ages 27.2 and 25.8 years)	–	5% (class I and II); 18% (class III)	–	Association between sella turcica bridging and manifest skeletal class III malocclusions.
Natsis et al. [14]	123 dry skulls	Greece	20–91 years	–	–	21.95%	Association between complete mineralisation and age and bilaterality.
Ota et al. [2]	72 CT angiograms for paraclinoid aneurysms	Japan	–	–	–	2.8%	Preoperative computed tomography is useful to detect variations in the anatomy around the ACP. When performing extradural anterior clinoidectomy.
Ozdogmus et al. [13]	50 autopsy specimens	Turkey	18–80 years	–	6%	–	Neurosurgical implications. No significant association between ossification and age.
Peker et al. [8]	80 dry skulls	Turkey	–	–	–	34.17%	Neurosurgical implications.
Scribante et al. [42]	78 lateral cephalometric radiographs	Italy	–	30% (controls)	13% (controls)	–	Higher incidence of sellar bridge formation in patients with dental anomalies.
Skrzat, Mroz, and Marchewka [19]	80 dry skulls	Poland	Adults	–	13.8%	–	Neurosurgical implications and effects upon internal carotid artery.
Suprasanna and Kumar [36]	54 CT angiograms	India	18–70 years	–	0.9%	–	Neurosurgical implications.
Aggarwal, Gupta, and Kumar [37]	67 dry skulls	India	–	5.2%	1.5%	6.7%	Neurosurgical implications.
Petrosphenoid ligament							
Current study	240 CT studies	UK	6–80 years	8.3%	2.5%	10.8%	–
Skrzat et al. [43]	1	Poland	–	–	–	–	Neurosurgical implications and possible role in abducens palsy.
Joo et al. [44]	10 cadaveric heads	Korea	–	–	–	25%	Anatomical features that may predispose to abducens palsy.
Inal et al. [16]	130 skull bases on CT	Turkey	20–78 years	9.8% (right); 9.8% (left)	2.3% (right); 2.9% (left)	–	Association between mineralisation and advancing age. Neurosurgical implications.
Özgür and Esen [11]	523 CT heads	Turkey	18–100 years	3.60%	2.20%	5.80%	Anatomical features that may predispose to abducens palsy.
Icke, Ozer, and Arda [45]	20 cadaveric heads	Turkey	–	–	–	5%	Neurosurgical implications. Variation in ligament morphology.
Aggarwal, Gupta, and Kumar [37]	67 dry skulls	India	–	3.0%	2.2%	5.2%	Neurosurgical implications.
Posterior petroclinoid ligament							
Current study	240 CT studies	UK	6–80 years	16.7%	1.2%	18.3% (Includes mixed 0.4%)	–
Cederberg et al. [23]	Lateral cephalometric radiographs of 255 subjects presenting for orthodontic evaluation	USA	8–76 years	23%	9%	32%	Very weak correlation with advancing age.
Inal et al. [16]	130 temporal bone CTs	Turkey	20–78 years	26.6% (right); 29.5% (left)	5.2% (right), 4.6% (left)	–	Neurosurgical implications. Anatomical features that may predispose to cranial nerve palsy.
Kimball et al. [18]	15 cadaveric head halves; 71 dry skulls	Grenada	68–93 years	13% (of cadaveric head halves)	20% (of cadaveric head halves)	9% skulls had large (> 2 mm) trigeminal protuberances	Neurosurgical implications. Potential role in trigeminal neuralgia.
Ozdede et al. [46]	290 cone beam CTs	Turkey	24–81 years	–	–	33.4% (calcification in general)	Male preponderance.
Patwardhan [47]	Case report	India	–	–	–	–	Anatomical features that may predispose to oculomotor palsy.
Sedghizadeh, Nguyen, and Enciso [48]	500 cone beam CTs	USA	13–82 years	–	–	8% (calcification in general bilateral only)	Common finding on dental cone beam CTs.
Skrzat et al. [49]	24 fixed specimens, 73 dry skulls (reviewed for ligament remnants)	Poland	–	–	–	1.4% (1 of 73 skulls)	Anatomy of non-calcified ligament and relationship with oculomotor nerve.
Wysiadecki et al. [50]	1	Poland	76 years	–	–	–	Association with oculomotor palsy.
Pterygospinous ligament							
Current study	240 CT studies	UK	6–80 years	12.5%	2.1%	17.1% (includes mixed 2.5%)	–
Goyal and Jain [51]	55 dried adult skulls and 20 sphenoid bones	India	–	14.67%	2.67%	17.33%	Implications for surgery and neural compression.
Shivanni and Yuvaraj Babu [52]	40 dry skulls	India	–	8%	–	8%	Surgical implications.
Yadav, Kumar, and Niranjan [53]	500 skulls	India	–	6.2%	4%	10.2%	Implications for neural compression.
Saran et al. [54]	50 dried skulls and 30 dried sphenoid bones	India	–	7.50%	1.25%	8.75%	Implications for surgery and neural compression.
Shinde, Mallikarjun, and Patil [55]	65 skulls	India	–	3.07%	–	3.07%	Implications for surgery and neural compression.
Tubbs et al. [56]	154 skulls	USA	–	0.645%	0.645%	1.3%	Implications for surgery.
Antonopoulou, Piagou, and Anagnostopoulou [57]	50 skulls	Greece	30–60 years	25%	2%	27%	Implications for neural impingement.
Nayak et al. 2007 [58]	416 dry skulls	India	–	3.84%	5.76%	9.61%	Phylogenetic origins and differences.
Das and Paul [59]	50 sphenoid bones	India	–	1%	0%	1%	Implications for surgery and neural compression.
von Lüdinghausen et al. [60]	100 skull bases. 54 halves of fixed cadaveric head and neck specimens	Japan and Germany	–	–	6%	–	Anatomical relationships on dissection. Phylogenetic differences.
Peuker, Fischer, and Filler [9]	1	Germany	–	–	–	–	Neural entrapment in a dissection specimen.
Tebo [61]	516 skulls	Skulls imported from India	–	33% (includes spines)	3.90%	–	Visibility on panoramic radiographs—can be mistaken for fracture.
Lepp and Sandner [22]	Not specified	Venezuela	–	–	–	–	Morphology anatomical review of the ligaments and implications for access to the foramen ovale.
Chouké [1]	n/a	USA	–	–	–	–	Technique modification for percutaneous access to the foramen ovale.
Chouké [62]	2745 skulls (in addition to skulls examined in 1946 paper)	USA	16–93 years	28.71%	5.46%	–	Implications for access to the foramen ovale.
Chouké [20]	1544 skulls	USA	16–101 years	–	6.28%	–	Anatomical description of the courses of the mineralised ligaments.
Shaw [63]	454 skulls	UK	Known in 80 cases: 18-60 years	11.7% partial or complete formation of a pterygospinous bar 16.1% (complete 4.4%)	4.4%	16.1%	Potential association with trigeminal neuralgia
Krmpotić-Nemanić et al. [7]	100 skulls; 50 isolated macerated sphenoid bones	Poland	Skulls 18–95 years; sphenoid bones 5–17 years	–	5%	–	Potential mechanisms for neural entrapment.
Ryu et al. [21]	142 skulls	Korea	Unknown	16.6%	1.4%	18%	Implications for neural impingement and surgical access.
Kamath and Kuberappa [64]	100 skulls	India	–	16%	1%	17%	Implications for neural impingement and surgical access.
Rosa et al. [65]	93 skulls (radiographed using the Hirtz axial technique)	Brazil	–	19.36%	8.61%	27.97%	Implications for neural impingement and surgical access.
Peker et al. [8]	452 skulls + mandibular nerves of 9 fixed cadavers	Turkey	–	–	5.50% (fixed); 8.8% (skulls)	–	Potential mechanism for neural entrapment.
Aggarwal, Gupta and Kumar [37]	67 dry skulls	India	–	6.7% (9 of 134 sides)	3.0% (4 of 134 sides)	9.7% (13 of 134 sides)	Implications for neural impingement and surgical access.
Pterygoalar ligament							
Current study	240 CT studies	UK	6–80 years	4.2%	1.3%	6.3% (includes mixed 0.8%)	–
Tubbs et al. [56]	154 skulls	USA	–	0.645%	0.645%	1.3%	Implications for surgical access.
Antonopoulou, Piagou, and Anagnostopoulou [57]	50 skulls	Greece	30–60 years	1%	7%	8%	Implications for neural impingement.
Lepp and Sandner [22]	Not specified	Venezuela	–	–	–	–	Morphology of the ligaments and implications for access to the foramen ovale.
Chouké [1]	n/a	USA	–	–	–	–	Technique modification for percutaneous access to the foramen ovale.
Chouké [62]	2745 skulls (in addition to skulls examined in 1946 paper)	USA	16–93 years	17.76%	5.94%	–	Anatomical characteristics of ligamentous mineralisation. No relationship with age.
Chouké [20]	1544 skulls	USA	16–101 years	–	10.30%	–	Anatomical characteristics of ligamentous mineralisation.
Shaw [63]	454 skulls	UK	Known in 80 cases: 18–60 years	–	0.67%	–	Relationship with trigeminal neuralgia.
Ryu et al. [21]	142 skulls	Korea	–	5.60%	2.80%	8.40%	Implications for surgical access and neural impingement.
Kamath and Kuberappa [64]	100 skulls	India	–	29%	1%	30%	Implications for surgical access and neural impingement.
Rosa et al. [65]	93 skulls (radiographed using the Hirtz axial technique)	Brazil	–	49.44%	12.91%	62.35%	Implications for neural impingement. Use of dedicated radiographic projections.
Peker et al. [8]	452 skulls + mandibular nerves of 9 fixed cadavers	Turkey	–	–	4.90% (fixed); 7.9% (skulls)	–	Potential mechanism for neural impingement.
Natsis et al. [66]	145 skulls	Greece	18–91 years	27.60%	4.10%	31.70%	Implications for neural impingement.
Pękala et al. [67]	Meta-analysis 25 studies	–	–	8.4% (overall pooled prevalence)	4.4% (overall pooled prevalence)	–	Meta-analysis.

*Includes elongation of the middle clinoid process

**Complete includes contact type

## Discussion

Mineralisation of skull base ligaments can occur as a result of an interplay between a broad range of factors, including genetics, metabolic abnormalities, and mechanical stress [68]. Such factors may explain de novo mineralisation later in life. However, the presence of ligamentous skull base mineralisation in children without an obvious inductive stimulus [12] may reflect developmental variation, which some have termed atavistic (i.e. representing evolutionary remnants) owing to the presence of similar ossified structures in non-human species [69].

It is clear from this study that mineralisation of skull base ligaments is a common finding (58.3%). In keeping with a suspected predominantly developmental origin, mineralisation was present in all age groups, although there was a nonsignificant trend towards an increased incidence with age. The association was however stronger for complete ligamentous mineralisation and varied with ligament type. In particular, the mean age of patients with posterior petroclinoid ligamentous mineralisation was higher than those with interclinoid or petrosphenoid mineralisation and was not observed in individuals aged 6–15 and 31–35 years. This finding likely reflects the nature of the posterior petroclinoid ligament, which is in fact a fold of dura mater (rather than a true ligament) that arises from the fixed portions of the tentorial incisura, and calcification of the dura is generally rarely seen in children [18, 19, 70]. There was no significant difference in the rate of ligamentous mineralisation amongst the largest ethnic groups included within the study; however, variance exists in the literature with higher rates of observed mineralisation in some (particularly Greek) populations, suggesting a potential genetic predisposition [14, 30, 57].

### Interclinoid and caroticoclinoid ligaments

Mineralised of these ‘sellar bridges’ was relatively commonly encountered within the studied population (22.1% and 17.5%, respectively). Whilst the incidence of caroticoclinoid mineralisation reflects the majority of prior studies (12–35.67% [2, 3, 5, 6, 8, 12, 26–29, 31–37]), there were some outliers [14, 30]. The incidence of interclinoid ligamentous mineralisation was higher in the current study than in many prior studies (4–11.8% [2, 12, 26, 27, 29, 36, 37, 39]), which may be secondary to the relatively long and exposed nature of the interclinoid ligament that could make it vulnerable to loss during the preparation of dry skulls. Indeed, a large Italian study of 300 CT scans of the head recorded incidences closer to the current study; furthermore, it corroborated our observation that mineralisation of the caroticoclinoid and interclinoid ligaments is not infrequently associated [32].

The clinical significance of mineralised interclinoid and caroticoclinoid ligaments arises primarily from their close relationships with the paraclinoid internal carotid artery (with the caroticoclinoid ligament potentially forming a solid ring around it) and cavernous sinus. In particular, the presence of ossified bars in these locations can make the extradural removal of the anterior clinoid process during clipping of paraclinoid aneurysms extremely difficult, requiring increased drilling and manipulation, which is accompanied by an increased potential risk of carotid rupture [2, 5, 14, 26, 31, 71]. Furthermore, these structures can complicate the excision of central skull base tumours where the internal carotid artery and cavernous sinus require exposure [2]. In addition, the presence of a completely mineralised caroticoclinoid ligament may alter the appearance of the middle clinoid process, which can be used as landmark for the anteromedial roof of the cavernous sinus and transition between the cavernous and clinoid segments of the internal carotid artery during endoscopic endonasal approaches to the pituitary gland [5, 6]. Furthermore, the presence of high-density calcification in the parasellar region may cause confusion on CT angiography if the viewer is unfamiliar with skull base ligamentous mineralisation; indeed, mineralisation of the interclinoid has been confused with para-posterior communicating artery aneurysm [38]. Finally, ‘sellar bridges’ have been associated with dental and other developmental abnormalities, including Gorlin-Goltz syndrome [26, 40, 42, 72, 73].

### Petrosphenoid ligament

This structure was amongst the least commonly mineralised skull base ligaments (10.8%), which is compatible with the published range of 5–25% [11, 16, 37, 43–45].

The clinical significance of petrosphenoid ligamentous mineralisation principally arises from its close relationship to the abducens nerve, which passes below it within Dorello’s canal [17]. For example, in the setting of raised intracranial pressure and uncal herniation, the mineralised ligament may protect the abducens nerve, but may present a noncompliant structure against which the oculomotor nerve may be compressed [16]. Furthermore, the passage of the abducens nerve beneath a densely mineralised ligament is postulated to have a role in abducens nerve palsy as it would create a noncompliant structure around the nerve, which would limit expansion in the setting of neural inflammation [11]. Finally, the petrosphenoid ligament is a helpful landmark during subtemporal-transtentorial-transpetrous approaches to the posterior and middle cranial fossae and its mineralisation may lead to the misidentification of anatomical localisation [16, 74].

### Posterior petroclinoid ligament (fold)

This structure was the second most commonly mineralised ligament (18.3%), which is higher than some studies of dry skulls (1.4–9%) [18, 49], but comparable to prior radiographic and CT studies [16, 23, 46]. This likely reflects the superiority of imaging in detecting fine calcified structures that may not be preserved in dry skulls.

The clinical significance of posterior petroclinoid ligament (or dural fold) mineralisation derives from its proximity to neural structures. In particular, in its course between the anterior petrous ridge to the posterior clinoid process, it forms the roof of the porus trigeminus and medial border of the oculomotor trigone (with the oculomotor nerve running over the ligament) [18]. In cases of mineralisation, Wysiadecki et al. found greater fixation of the dural sheath of the oculomotor nerve, which may increase the risk of neural injury during intraoperative manipulation, and prior division with an appropriate instrument may be required [16, 50]. It may also increase the risk of oculomotor neural injury following relatively insignificant head trauma, as a result of compression of the nerve against a noncompliant ligament [10, 47]. Finally, there has been speculation that compression of the trigeminal nerve may occur in the setting of an extensively mineralised posterior petroclinoid ligament and may be considered for those in whom prior microvascular decompression has failed [18, 75].

### Pterygospinous and pterygoalar ligaments

In the current study, these structures were found to be mineralised in 17.1% and 6.3% (pterygospinous and pterygoalar ligaments, respectively) of patients. The published rate of ligamentous mineralisation is variable (1–27.97% for the pterygospinous ligament [7, 8, 20, 21, 51–58, 60–65] and 1.3–62.35% for the pterygoalar ligament [8, 20–22, 56, 57, 62–66]), but the latter was comparable to a recent meta-analysis [67].

The clinical significance of pterygospinous and pterygoalar ligamentous mineralisation arises from their capacities to form barriers to surgical access as well as their close relationship to neural structures. Although both ligaments are in close proximity anatomically, they are distinct in their courses, most notably posteriorly, with the pterygospinous ligament (a thickening of the interpterygoid aponeurosis) attaching to the spine of the sphenoid and the pterygoalar ligament (a thickening of the lateral interpterygoid or pterygotemporomaxillary aponeurosis) attaching more laterally to the undersurface of the sphenoid [22]. Furthermore, whilst both ligaments attach to the lateral pterygoid plate anteriorly, the pterygoalar ligament attaches more superiorly, at the level of the root [20]. This is particularly relevant for access to the foramen ovale for percutaneous rhizotomy or cavernous sinus biopsy where a mineralised pterygoalar ligament can create a wall-like barrier lateral to the foramen ovale, making percutaneous access difficult or even impossible, particularly via a trans-zygomatic approach [1, 21, 51, 64, 66, 76]. In addition, mineralisation of either ligament may impede trans-zygomatic exploration of the external skull base as well as the parapharyngeal or retropharyngeal spaces [21, 60].

Following the descent of the mandibular division of the trigeminal nerve through the foramen ovale, it undergoes branching. Some of these pass through the foramina created by the mineralised pterygospinous and pterygoalar ligaments. In particular, branches to the tensors tympani and veli palatini and medial pterygoid can pass through the foramen of Civinini and motor branches to the temporal, buccinator lateral pterygoid, and sometimes masseter muscles may pass through the foramen created by the pterygoalar ligament [7, 20, 22]. However, the association with neural branches is variable; indeed, von Lüdinghausen et al. described four potential branching patterns (A–D) in relation to a mineralised pterygospinous ligament with lateral displacement of the branches to the temporalis, masseter, and pterygoid muscles being most common and medial displacement of the branches being least common [60]. Others have described further variations, such as division of the lingual nerve into an anterior and posterior division by a mineralised ligament, which can increase the risk of entrapment [77]. Entrapment may also arise when the lingual nerve passes between an ossified pterygospinous ligament and the medial pterygoid muscle [9, 67, 78]. In addition, Krmpotić-Nemanić et al. noted that various types of lateral pterygoid plate enlargement (including complete ossification of the pterygospinous ligament) resulted in the displacement of the lingual and inferior alveolar branches resulting in fixation and increased risk of compression [7]. It is also suggested that a mineralised pterygospinous ligament may potentially cause the compression of other branches of the mandibular nerve (auriculotemporal nerve in particular), leading to periauricular sensory or parotid glandular secretomotor symptoms [64, 66, 76].

### Limitations

Whilst noncontrast CT provides excellent delineation of mineralised structures, it does not allow for the detailed visualisation of soft tissue anatomy such as nerves and blood vessels that may be affected by ligamentous mineralisation. In the future, MRI may be useful in determining the precise relationships between mineralised ligaments and local cranial nerves. In addition, given the retrospective nature of the study, only limited clinical data was available; therefore, it is not known whether any of the cases included suffered symptoms in relation to ligamentous mineralisation.

### Conclusion

The presence of ligamentous skull base mineralisation is a relatively common phenomenon on CT. These structures can present barriers to minimally invasive surgical access to the infratemporal fossa and increase the risk of neurovascular injury at the central skull base. Furthermore, ligamentous mineralisation has been implicated in neural entrapment. Therefore, knowledge of these structures is of great importance to avoid undesirable complications.

## Abbreviations

CT: Computed tomography
ICA: Internal carotid artery
PACS: Picture archiving and communication system
